# The Relationship between Physical Exercise and Cognitive Function in Korean Middle Aged and Elderly Adults without Dementia

**DOI:** 10.3390/ijerph17238821

**Published:** 2020-11-27

**Authors:** Youngseung Koh, Yeonsu Oh, Haesung Park, Woorim Kim, Eun-Cheol Park

**Affiliations:** 1 Yonsei University College of Medicine, Seoul 03722, Korea; yskoh361@yonsei.ac.kr (Y.K.); nasaoh@naver.com (Y.O.); comet129@naver.com (H.P.); 2 Division of Cancer Control & Policy, National Cancer Control Institute, National Cancer Center, Gyeonggi-do 10408, Korea; 3 Institute of Health Services Research, Yonsei University, Seoul 03722, Korea; 4 Department of Preventive Medicine, Yonsei University College of Medicine, Seoul 03722, Korea

**Keywords:** physical exercise, exercise duration, exercise frequency, cognitive function, middle aged, elderly

## Abstract

This study investigated the association between physical exercise and cognitive function in Koreans aged 45 years or above without dementia. Data from the 2006 to 2018 Korean Longitudinal Study of Aging (KLoSA) were used. The general characteristics of the study population were investigated using analysis of variance (ANOVA). The association between total exercise time per week and cognitive function, measured based on the Mini-Mental State Examination (MMSE) scores, was investigated using the generalized estimating equation (GEE) model. Subgroup analysis was conducted based on age, educational level, and marital status. A total of 8888 participants were investigated, of which 5173 (58.2%) individuals did not exercise regularly. Among participants who did exercise, 676 (7.6%) individuals were categorized into the Q1, 1157 (13.0%) into the Q2, 908 (10.2%) into the Q3, and 974 (11.0%) into the Q4 group. The mean MMSE score was 26.81 ± 3.17. Compared to the ‘no’ exercise group, better MMSE scores were found in the Q1 (β: 0.3523, *p* ≤ 0.0001), the Q2 (β: 0.2011, *p* ≤ 0.0001), the Q3 (β: 0.4075, *p* ≤ 0.0001), and the Q4 groups (β: 0.3144, *p* ≤ 0.0001) after adjustment. The magnitude of this association was stronger in participants aged 65 years or above and in single or separated individuals. The findings of this study confirm a positive association between physical exercise and MMSE scores in the middle aged and elderly.

## 1. Introduction

Dementia is a degenerative disease characterized by noticeable cognitive decline, often diagnosed when normal social and occupation functioning of an individual is compromised due to cognitive impairment [1]. Age is a strong risk factor for dementia because it is a degenerative disease, making dementia an important issue in many East Asian countries that face a rapidly aging population. Issues on dementia are particularly significant in South Korea because it is one of the world’s most rapidly aging countries [2]. The prevalence of dementia shows an increasing trend, with it estimated at 5.0% and the prevalence of mild cognitive impairment at 27.0% [3]. Unsurprisingly, the number of dementia patients in expected to continuously increase in the future, because patient numbers tend to nearly double with each 5.8-year increase in average age [4].

Although the causes of dementia are not fully understood due to its complex and multifactorial nature, features such as eating habits, mental diseases including depression, genetics, and individual lifestyle have been reported as common risk factors [5,6,7,8,9,10,11,12]. Specifically, physical exercise is a well-known and modifiable risk factor for many age-related diseases, including dementia [13]. In fact, the positive association between physical exercise and cognitive function in the middle-aged and elderly has been well established. A previous study presented that aerobic and balance exercise is beneficial in improving cognitive performance in middle-aged adults [14]. Likewise, physical exercise has been related to better cognitive and brain health in the elderly [15]. However, despite such importance, few studies have investigated this relationship in East Asian countries because dementia has only gained significant importance recently compared to many Western countries with an already aged population. Furthermore, the few Korean studies present on this topic were relatively small-scaled or targeted only a specific group of individuals [16,17].

Therefore, the aim of this study was to investigate the association between physical exercise and cognitive status in middle and older aged Korean adults without dementia using the Mini-Mental State Exam (MMSE), a simple scale developed to test cognitive ability. The hypothesis was that individuals who regularly exercise will score better on the MMSE scale than their physically inactive counterparts. Additionally, subgroup analysis was conducted based on age, educational level, and marital status to further examine how these factors can potentially interplay in the main relationship stated above.

## 2. Materials and Methods

### 2.1. Study Population

This study used data from the 2006 to 2018 Korean Longitudinal Study of Aging (KLoSA). The KLoSA is a biennial panel study conducted by the Korean Employment Information Service. Individuals aged 45 years or above at the 2006 baseline were randomly selected nationwide. Households residing on islands, including Jeju island, were excluded from the sampling frame. Institutionalized individuals were also excluded. The target population was selected using a stratified, multi-stage area probability sampling method to obtain a nationally representative sample of individuals aged 45 years or above. Region was first stratified into urban and rural areas based on the 15 administrative districts of South Korea, which was further categorized into apartment and ordinary housing (houses, townhouses, multifamily houses) areas [18]. Of the 1000 sample districts, each of the 15 administrative districts were allocated with 15 sample districts (225 total), and the remaining 775 sample districts were assigned based on population size [18].

A total of 10,254 individuals were included in the 2006 data [19]. The average follow-up rate was 77.6% (2008: 86.6%; 2010: 81.7%; 2012: 80.1%; 2014: 80.4%; 2016: 79.6%; 2018: 78.8%) [19]. Years of observation were 2006, 2008, 2010, 2012, 2014, 2016, and 2018. Information was collected through face-to-face interviews conducted using the Computer Assisted Personal Interviewing (CAPI) technique. Missing values of the 20 major variables were replaced using the multiple imputation method [20]. The reported proportion of missing data were between 10% to 20% for income or wealth related variables and less than 5% for the other variables in the KLoSA.

**Figure 1:**
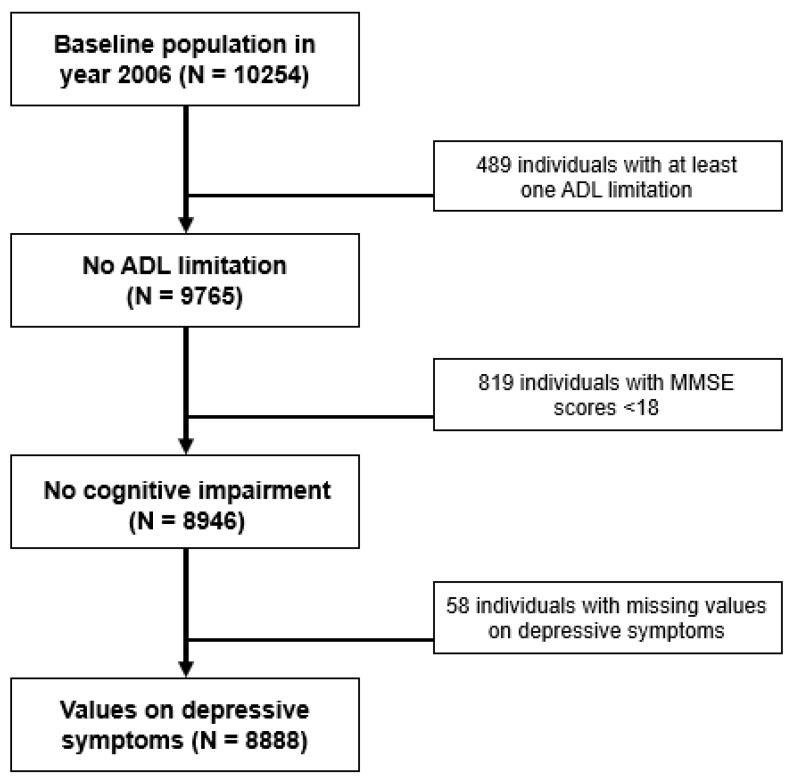
Study population selection process.

Details on the study population are shown in Figure 1. Of the 10,254 individuals included in the 2006 data, 489 participants incapable of performing at least one of the basic Activities of Daily Living (ADL) and 819 participants with MMSE scores below 19 were excluded because those with poor basic ADL performance or dementia may be unable to perform regular physical exercise. Additionally, 58 individuals with missing values on depression measured using the Centre for Epidemiological Studies Depression (CES-D) were not included. This led to the final baseline study population of 8888 individuals.

### 2.2. Outcome Measure

The outcome measure of this study was cognitive function, measured using the Korean-Mini-Mental Status Examination (K-MMSE). The MMSE is a known index used to examine and diagnose patients with dementia [21]. The K-MMSE has previously been validated in a comparative study. A K-MMSE score over 24 points implies ‘definite normal’ status, 20–23 points implies ‘cognitive decline’, and below 19 points implies ‘dementia [21]’.

### 2.3. Physical Exercise

Physical exercise was measured based on the following three items: total exercise time per week (minutes per week), duration per exercise (minutes per exercise), and exercise frequency per week (days per week). The KLoSA collects information on exercise frequency and duration, measured based on the recommended level of physical exercise performed regularly [22]. This is a standardized question utilized widely in previous literature to outline self-reported exercise [23]. Total exercise time per week, the main variable of interest, was calculated based on exercise duration and frequency. Individuals exercising regularly were categorized into quartiles. The ranges were set at 120, 240, and 420 min per week.

### 2.4. Covariates

Different demographic, socioeconomic, and health behavior-related covariates were incorporated in this study. The included covariates were sex (male or female), age (45–64 years or 65 years or above), age^2^, household income (quartiles), educational level (high school or below or college or above), marital status (single or separated, or married and cohabiting with spouse), depressive symptoms (no or yes), chronic diseases (none, one, or two or above), smoking status (no or yes), perceived health status (poor or fair), and region (rural areas, small to medium sized cities, or large cities). Age^2^ was included as a covariate to account for the strong association between age and the dependent variable [24]. Chronic diseases included diabetes, hypertension, and chronic obstructive pulmonary disease (COPD). Depressive symptoms were measured using the 10-item Centre for Epidemiological Studies Depression (CES-D 10) scale. The CES-D 10 has been widely utilized to screen for depressive symptoms in older adults [25].

### 2.5. Statistical Analysis

One-way analysis of variance (ANOVA) was used to investigate the general characteristics of the study population. The association between physical exercise and MMSE scores were analyzed using the generalized estimating equation (GEE) model, an extension of the quasi-likelihood approach used to analyze longitudinal correlated data in the form of counts [26]. The GEE model takes into account time variation and the correlations between repeated measurements found in a longitudinal study design [26]. The GEE model was applied because the KLoSA data are longitudinal in design and contain repeated measurements, inferring the need to account for the correlation within subjects. Total exercise time per week was the main interesting variable, although analysis was also conducted for the other two items on physical exercise based on separate models. Model 1 depicts the results of the unadjusted analysis and Model 2 that of the adjusted analysis. Subgroup analysis was conducted based on age, educational level, and marital status. All *p*-values were two-tailed, and the level of significance was set at 0.05. All analyses were conducted using the SAS version 9.4 (SAS, Cary, NC, USA).

### 2.6. Ethical Concerns

This paper complies with the ethical standards of the relevant national and institutional committees on human experimentation and the Helsinki Declaration of 1975. This study used open, secondary data in which all personal information was anonymized and unidentifiable, therefore attainment of an informed consent was waived. Access to the KLoSA data was acquired after upon its approval by the Institutional Review Board (IRB) of the Korea National Institute for Ethics Policy (P01-201909-22-002). The KLoSA data are also part of the Official Statistics collected and utilized based on the Statistics Act of Korea.

## 3. Results

The characteristics of the study participants at baseline are shown in Table 1. Of the 8888 participants, 5173 (58.2%) individuals did not exercise regularly. Among individuals who did exercise regularly, 676 (7.6%) participants were included into the Q1 group, 1157 (13.0%) into the Q2 group, 908 (10.2%) into the Q3 group, and 974 (11.0%) into the Q4 group. Individuals who did not exercise had the poorest MMSE scores. The mean MMSE scores at baseline were 26.81 ± 3.17, as displayed in Table 2.

The results of the GEE analysis on the association between MMSE scores and physical exercise (total exercise time per week, duration per exercise, and exercise frequency per week) are presented in Table 3. Model 1 shows the results of the unadjusted analysis and Model 2 shows that of the adjusted analysis. MMSE scores tended to escalate as total exercise time per week increased in both models. Without adjustment, individuals in the Q1 group (β: 0.5261, *p* ≤ 0.0001), the Q2 group (β: 0.3244, *p* ≤ 0.0001), the Q3 group (β: 0.5815, *p* ≤ 0.0001), and the Q4 group (β: 0.5626, *p* ≤ 0.0001) showed better MMSE scores than participants who did not exercise. After adjustment, compared to the ‘no’ exercise group, better MMSE scores were found in the Q1 (β: 0.3523, *p* ≤ 0.0001), Q2 (β: 0.2011, *p* ≤ 0.0001), Q3 (β: 0.4075, *p* ≤ 0.0001), and Q4 groups (β: 0.3144, *p* ≤ 0.0001) groups. Analysis on the relationship between MMSE scores and duration per exercise and exercise per frequency showed similar tendencies, with MMSE scores showing a positive correlation with exercise length and frequency.

The results of the subgroup analysis on the association between MMSE scores and physical exercise by age, education level, and marital status are shown in Table 4. The tendencies of the main findings were generally maintained in all groups. However, the magnitude of increase was more profound in older individuals aged 65 years or above than those aged between 45 and 64 years. The degree of association was also stronger in single or separated individuals compared to married participants cohabiting with their spouse. The *p*-values for interaction showed significance for all three variables, namely age (*p* ≤ 0.0001), education level (*p* = 0.0377) and marital status (*p* = 0.0002).

## 4. Discussion

The results of this study confirm an association between physical exercise and MMSE scores in the Korean middle-aged and elderly population without dementia. Individuals not exercising regularly showed comparatively poorer MMSE scores compared to the ‘yes’ exercise group. The highest level of increases was found in the Q3 exercise group, followed by the Q1, Q4, and Q2 groups. The positive correlations between physical exercise and cognitive function were maintained when examining physical exercise based on duration per workout (minutes per exercise) and frequency (days per week).

The presented findings are in accordance with previous studies, which have demonstrated that physical exercise is related to higher cognitive performance in adults [27]. A previous meta-analysis confirmed that physical exercise can reduce risk of cognitive deficits, supporting the results of this study [28]. Previous studies have also revealed the positive influence of exercise duration on cognitive debilitation [29]. Additionally, exercise frequency has also been associated with cognitive functioning, with studies reporting that even regular or light exercise can reduce the risk of developing dementia in the elderly [30]. Considering the high proportion of middle aged and elderly individuals reporting to not exercise regularly in South Korea, the results offer important insights by revealing the potential benefits of physical exercise.

In this study, individuals regularly exercising clearly showed better cognitive performance than those not conducting exercise. However at the same time, a dose-response relationship was not found because compared to the ‘no’ exercise group, the Q3 group showed the highest level of increase in MMSE score, followed by the Q1, Q4, and Q2 groups. Many previous studies on this topic have reported a similar inclination. Specifically, despite the establishment of a strong relationship between physical exercise and cognitive status, reports on its dose–response relationship have shown mixed results because they do not show a stepwise increase based on exercise frequency or duration [31,32]. Others suggest various levels of exercise quantity or quality as a threshold level to prevent cognitive decline [33,34]. As such, future targeted studies are needed to verify the presence of a dose–response relationship on this topic.

The results of the subgroup analysis show that the relationship between physical exercise and cognitive function is maintained regardless of age, educational level, and marital status. However, a stronger degree of association was found in the elderly aged 65 years or above. The assistive benefits of exercise on cognitive function may have been more profound in older aged adults because physical and cognitive functioning are known to decline with age. Moreover, the relationship found was also more significant in single or separated individuals compared to married individuals cohabiting with their spouse. The results are understandable, considering that social factors, such as cohabitation status, have been previously associated with cognitive decline [35,36]. In fact, cohabitation status has been suggested as a risk factor for cognitive deterioration, with studies reporting that the elderly living alone or with a non-spouse family member may be more vulnerable to physical and cognitive deterioration [37,38,39]. This may result due to the loss of a spouse leading to disruptions in carrying out daily activities or routines [40].

This study has some limitations. Firstly, exercise intensity could not be taken into account due to data limitation. The KLoSA data only provide information on exercise frequency per week and exercise duration per workout. However, the KLoSA basic analysis report states that exercise duration and frequency was measured based on the recommended level of physical exercise individuals responded to participate in the survey [22]. Thus, a certain level of homogeneity was ensured in the data utilized. Secondly, although cognitive status was measured based on the previously validated K-MMSE, assessment made based on its cut-off value does not necessarily imply a clinical diagnosis of dementia. Thirdly, although this study excluded individuals with ADL limitations or dementia because they may have a reduced level of physical competence, the possibility of reverse causality cannot be completely ruled out. Lastly, although this study did adjust for various confounding variables, partial residual confounding may still have been present. However, despite the limitations stated above, this study is unique in that it examined the relationship between physical exercise and cognitive function using a large, nationally representative sample of South Korean middle-aged and elderly adults. The findings offer important insights because they reveal that participating in physical exercise itself can help preserve cognitive functioning.

## 5. Conclusions

The findings of this study confirm a positive association between participating in physical exercise and better MMSE scores in the middle aged and elderly without dementia. This tendency was maintained regardless of age, educational level, and marital status. The degree of this relationship was magnified in individuals aged 65 years or above and in single or separated individuals. The results highlight the importance of promoting physical exercise to prevent cognitive declines in older aged adults, which is important considering the rapidly aging population found in many countries.

## Figures and Tables

**Table 1:** General characteristics of the study population.

	MMSE Scores					
	N	(%)	Mean ± SD			*p*-Value
Total Exercise Time per Week (min/week)						
None	5173	(58.2)	26.43	±	3.38	<0.0001
Q1	676	(7.6)	27.47	±	2.77	
Q2	1157	(13.0)	27.23	±	2.85	
Q3	908	(10.2)	27.52	±	2.65	
Q4	974	(11.0)	27.21	±	2.75	
Duration per exercise (minutes/exercise)						
None	5173	(58.2)	26.43	±	3.38	<0.0001
Short (≤59)	1220	(13.7)	26.96	±	2.98	
Moderate (60–119)	1822	(20.5)	27.45	±	2.70	
Long (≥120)	673	(7.6)	27.73	±	2.44	
Exercise frequency per week (days/week)						
Not frequent (0)	5173	(58.2)	26.43	±	3.38	<0.0001
Moderate (1–3)	1472	(16.6)	27.68	±	2.54	
Frequent (4–7)	2243	(25.2)	27.11	±	2.88	
Sex						
Male	4081	(45.9)	27.39	±	2.79	<0.0001
Female	4807	(54.1)	26.32	±	3.39	
Age						
45–64	3196	(36.0)	28.14	±	2.25	<0.0001
65 or above	2596	(29.2)	27.08	±	2.86	
Household income						
Low	2245	(25.3)	25.88	±	3.41	<0.0001
Low-middle	2314	(26.0)	26.40	±	3.32	
Middle-high	2115	(23.8)	27.15	±	2.90	
High	2214	(24.9)	27.85	±	2.59	
Educational level						
High school or below	7796	(87.7)	26.56	±	3.23	<0.0001
College or above	1092	(12.3)	28.62	±	1.91	
Marital status						
Single or separated	1622	(18.2)	25.31	±	3.67	<0.0001
Married and cohabiting with spouse	7266	(81.8)	27.14	±	2.95	
Depressive symptoms						
No	6417	(72.2)	27.25	±	2.94	<0.0001
Yes	2471	(27.8)	25.66	±	3.44	
Chronic diseases						
None	5999	(67.5)	27.12	±	3.04	0.8421
1	2285	(25.7)	26.23	±	3.30	
2 or above	604	(6.8)	25.89	±	3.48	
Smoking status						
No	7079	(79.6)	26.66	±	3.23	0.9359
Yes	1809	(20.4)	27.40	±	2.83	
Perceived health status						
Fair	4580	(51.5)	27.72	±	2.65	<0.0001
Poor	4308	(48.5)	25.84	±	3.38	
Region						
Rural areas	4044	(45.5)	27.10	±	3.05	<0.0001
Small to medium sized cities	2941	(33.1)	26.83	±	3.15	
Large cities	1903	(21.4)	26.16	±	3.36	
Total	8888	(100.0)	26.81	±	3.17	

**Table 2:** Mean MMSE scores by survey year.

Year	Mean ± SD		
2006	26.81	±	3.17
2008	26.61	±	3.20
2010	26.61	±	3.28
2012	26.77	±	3.23
2014	26.61	±	3.39
2016	26.87	±	3.31
2018	26.72	±	3.39

**Table 3:** The association between MMSE scores and physical exercise.

	Model 1 *			Model 2 *		
	β	SE	*p*-Value	β	SE	*p*-Value
Total exercise time per week (minutes/week)						
None	Ref			Ref		
Q1	0.5261	0.0446	<0.0001	0.3523	0.0436	<0.0001
Q2	0.3244	0.0448	<0.0001	0.2011	0.0433	<0.0001
Q3	0.5815	0.0415	<0.0001	0.4075	0.0399	<0.0001
Q4	0.5626	0.0473	<0.0001	0.3144	0.0463	<0.0001
Duration per exercise (minutes/exercise)						
None	Ref			Ref		
Short (≤59)	0.2840	0.0426	<0.0001	0.2903	0.0414	<0.0001
Moderate (60–119)	0.5742	0.0349	<0.0001	0.3576	0.0337	<0.0001
Long (≥120)	0.7321	0.052	<0.0001	0.2662	0.0508	<0.0001
Exercise frequency per week (days/week)						
Not frequent (0)	Ref			Ref		
Moderate (1–3)	0.5433	0.0377	<0.0001	0.2713	0.0364	<0.0001
Frequent (4–7)	0.4692	0.0352	<0.0001	0.3552	0.0338	<0.0001
Sex						
Male				Ref		
Female				−0.8513	0.0461	<0.0001
Age						
45–64				Ref		
65 or above				0.2269	0.0421	<0.0001
Age^2^				−0.0007	0.0000	<0.0001
Household income						
Low				Ref		
Low-middle				0.3337	0.0419	<0.0001
Middle-high				0.6016	0.043	<0.0001
High				0.8689	0.0445	<0.0001
Educational level						
High school or below				Ref		
College or above				0.0257	0.0511	0.6150
Marital status						
Single or separated				Ref		
Married and cohabiting with spouse				0.3077	0.0571	<0.0001
Depressive symptoms						
No				Ref		
Yes				−0.6813	0.0303	<0.0001
Chronic diseases						
None				Ref		
1				−0.0430	0.0414	0.2991
2 or above				−0.1125	0.0689	0.1026
Smoking status						
No				Ref		
Yes				−0.0501	0.0482	0.2988
Perceived health status						
Poor				Ref		
Fair				0.4301	0.0296	<0.0001
Region						
Rural areas				Ref		
Small to medium sized cities				−0.2952	0.0461	<0.0001
Large cities				−0.5011	0.0537	<0.0001

* Total exercise time per week, duration per exercise, and exercise frequency per week analyzed in three separate models, adjusted for the same variables; Results of the covariates are from the analysis on total exercise time per week. SE, standard error.

**Table 4:** Results of the subgroup analysis.

	MMSE Scores *					
	β	SE	*p*-Value	β	SE	*p*-Value
Age	Middle-aged (45–64)			Elderly (65+)		
Total exercise time per week (min/week)						
None	Ref			Ref		
Q1	0.2553	0.0479	<0.0001	0.5403	0.0778	<0.0001
Q2	0.1600	0.0494	0.0012	0.2442	0.0727	0.0008
Q3	0.2115	0.0465	<0.0001	0.6488	0.0674	<0.0001
Q4	0.1023	0.0565	0.0701	0.5267	0.0727	<0.0001
Education level	Middle school or below			High school or above		
Total exercise time per week (min/week)						
None	Ref			Ref		
Q1	0.3441	0.0458	<0.0001	0.4271	0.122	0.0005
Q2	0.1781	0.045	<0.0001	0.4512	0.1296	0.0005
Q3	0.4161	0.0414	<0.0001	0.1059	0.1338	0.4287
Q4	0.3221	0.0487	<0.0001	0.1383	0.138	0.3164
Marital status	Single or separated			Married and cohabiting with spouse		
Total exercise time per week (min/week)						
None	Ref			Ref		
Q1	0.3593	0.1189	0.0025	0.3460	0.0461	<0.0001
Q2	0.2207	0.1154	0.0559	0.1923	0.0458	<0.0001
Q3	0.4616	0.1203	0.0001	0.3906	0.0414	<0.0001
Q4	0.5174	0.1374	0.0002	0.2765	0.0484	<0.0001

* Adjusted for sex, age, household income, education level, marital status, depressive symptoms, chronic diseases, smoking status, perceived health status, and region. SE, standard error.
